# Influenza vaccination in pregnancy: vaccine uptake, maternal and healthcare providers’ knowledge and attitudes. A quantitative study

**DOI:** 10.3399/bjgpopen18X101599

**Published:** 2018-08-08

**Authors:** Tina Barrett, Edel McEntee, Richard Drew, Fiona O’Reilly, Austin O’Carroll, Aisling O’Shea, Brian Cleary

**Affiliations:** 1 Medical Student, Medicine Department, Royal College of Surgeons in Ireland, Dublin, Ireland; 2 Clinical Pharmacist, Pharmacy Department, Rotunda Hospital, Dublin, Ireland; 3 GP Graduate, General Practice, North Dublin City GP Training Programme, Dublin, Ireland; 4 Consultant Microbiologist, Microbiology Department, Rotunda Hospital, Dublin, Ireland; 5 Programme Director, GP Training Scheme, North Dublin City GP Training Programme, Dublin, Ireland; 6 Head of the NDCGP Training Scheme, North Dublin City GP Training Programme, Dublin, Ireland; 7 GP Graduate, GP Training Scheme, North Dublin City GP Training Programme, Dublin, Ireland; 8 Chief Pharmacist, Pharmacy Department, Rotunda Hospital, Dublin, Ireland

**Keywords:** influenza vaccine, infectious pregnancy complications, inactivated vaccines

## Abstract

**Background:**

Influenza during pregnancy is a potentially life threatening illness. There are limited data on influenza vaccination uptake and determinants of uptake in Irish obstetric populations.

**Aim:**

To determine the uptake of influenza vaccination during pregnancy; determinants of vaccination uptake; knowledge, attitudes, and concerns of postnatal women; and knowledge and attitudes of healthcare professionals (HCPs) surrounding vaccination.

**Design & setting:**

A quantitative study of postnatal women attending the Rotunda Hospital, a tertiary referral maternity hospital in Dublin, Ireland. A separate quantitative study conducted by the North Dublin City GP Training Programme surveyed GPs, pharmacists, and Rotunda Hospital clinical staff.

**Method:**

A paper-based survey was distributed to postnatal women. HCPs completed the survey via the online tool Survey Monkey.

**Results:**

330 patient surveys were disseminated, with a 60.0% response rate. Of 198 responders, 109 (55.1%) were vaccinated against influenza. Non-professionals were less likely to be vaccinated (adjusted odds ratio [aOR] 0.29, 95% confidence interval [CI] = 0.09 to 0.89). Vaccination in previous pregnancy (aOR 5.2, 95% CI = 1.69 to 15.62) and information from an HCP were strongly associated with vaccination (aOR 12.8, 95% CI = 2.65 to 62.5). There was a 20.2% (*n* = 1180) response rate among HCPs. More GPs felt that it was their role to discuss vaccination (92.9%; *n* = 676), and offer to vaccinate women (91.7%; *n* = 666) than any other HCP.

**Conclusion:**

Provision of information about the importance of vaccination against influenza and pertussis during pregnancy by HCPs and their consistent recommendations in support of vaccination were key determinants of vaccine uptake during pregnancy. The sociodemographic determinants of a woman’s vaccination status should be addressed in health promotion campaigns. Education of HCPs may address knowledge gaps surrounding vaccination.

## How this fits in

A lack of data on the uptake of influenza and pertussis vaccination in Irish obstetric healthcare settings prompted this study. Similar Irish studies have collected data over a shorter period of time. This particular study has collected data over a 6-month period, from women that were pregnant throughout the influenza season.

It is hoped the method of data collection from postnatal women has given the most accurate view of vaccination uptake among pregnant women in Ireland.

Future health promotion campaigns may use the information in this study to address the concerns and awareness of pregnant women, and to improve vaccination uptake.

## Introduction

Influenza during pregnancy is a potentially life-threatening illness. Vaccination against influenza was an important focus of the 2009–2012 MMBRACE report, which reported that 1 in 11 maternal deaths in the UK and Ireland were caused by influenza.^1^ Pregnant women are listed as a high priority group for influenza vaccination.^2^

Physiological changes of pregnancy may predispose pregnant women to complications including hospitalisation, ventilation, and preterm birth.^3^

In Ireland, the inactivated influenza vaccine is recommended for all pregnant women at any stage during pregnancy.^3,4^ Maternal influenza vaccination during pregnancy may also confer benefits to infants in the first months of life.^5^

In the Rotunda Hospital, pregnant women are routinely asked about their vaccination status at their booking interview. Women are also reminded to receive the flu vaccine in a text message reminder for antenatal appointments.

In Ireland, the national Health Service Executive (HSE) conducts an annual influenza vaccination campaign and information is distributed to GPs, pharmacies, and hospitals.^4^

At present in Ireland, there are few current data on the number of women vaccinated against influenza or pertussis during pregnancy. A Health Protection and Surveillance Centre audit in 2013 reported that 18% of women were vaccinated against influenza and 6.4% vaccinated against pertussis.^6^ The influenza vaccination rate among women in the antenatal clinic of an Irish maternity hospital was reported as 39.1% in 2016.^7^ A high influenza vaccine uptake rate of 2009 Influenza A/H1N1 vaccine among pregnant women was achieved during the 2009 pandemic, with >70% of pregnant women vaccinated in the weeks after initiation of the vaccination programme.^8^

Similar studies have investigated uptake of influenza vaccine among pregnant women, and the determinants of vaccine uptake.^8–11^ An association has been found between knowledge of the protective benefits of vaccination during pregnancy and receiving the influenza vaccine.^12^ International studies have demonstrated that women’s understandings of the risk of complications caused by influenza, and receiving a recommendation to receive the vaccine from an HCP, have strong associations with receiving the vaccine.^9,10,13^ The majority of women concerned about the safety of vaccination would be vaccinated if their HCP recommended it.^10^ Negative media reports on possible associations between the influenza vaccine and adverse outcomes have been described as barriers to vaccination uptake.^14^

Social inequalities of vaccine uptake are apparent for other vaccines such as the measles, mumps and rubella (MMR) vaccine.^15^ Immigrant women and those of low socioeconomic status were less likely to be vaccinated during the H1N1 pandemic.^16^

This study aimed to assess influenza vaccination in pregnancy from maternal and HCPs’ perspectives. Objectives included an assessment of vaccine uptake and determinants of uptake. Knowledge, attitudes, and concerns of postnatal women surrounding vaccination during pregnancy were assessed along with uptake of pertussis vaccine during pregnancy. HCPs knowledge, attitudes, and behaviours regarding influenza vaccination during pregnancy were assessed.

## Method

### Survey of postnatal women

#### Survey design

The survey focused on women on postnatal wards during and after the peak influenza season to estimate maximal uptake.^17^

A standardised questionnaire was developed and pilot-tested by the research team (further information available from the authors on request). Research Ethics Committee approval was received in November 2015. No incentives were offered to women for taking part.

#### Sample size and recruitment

A sample size of 300 women was proposed in order to ensure sufficient representation of different socioeconomic groups. Exclusion criteria included: age <18 years, those with non-live births, and those who delivered babies at <24 weeks’ gestation. Recruitment took place between January 2016 and June 2016.

Survey forms were distributed to a convenience sample of 330 postnatal women. Women were informed that the survey was entirely optional and anonymous. They were given an information leaflet and a survey. They were verbally consented to participate. Survey forms were collected at a later point on the same day by a member of the research team or were returned to a midwife. Throughout the study, the numbers of refusals were not routinely recorded. In the initial weeks of the study, refusal rates were between 5–10% of patients approached.

#### Data analysis

Univariable logistic regression analysis was carried out to assess the association between maternal demographics and vaccination status. Forward stepwise logistic regression was used to generate aORs for the association between maternal characteristics and influenza vaccination status. Data were analysed using SPSS (version 23).

### HCP survey

#### Survey design

HCPs were asked to complete an online survey (further information available from the authors on request) distributed via professional bodies or hospital email accounts. Questions focused on knowledge of the consequences of influenza during pregnancy, and their attitudes and recommendations for vaccination.

#### Sample size and recruitment

The Irish College of General Practitioners (ICGP), the Pharmaceutical Society of Ireland (PSI), and the Rotunda Hospital facilitated the distribution of questionnaires to all GPs, and third and fourth year GP registrars; 50% of the pharmacists on the PSI register; and HCPs based at the Rotunda Hospital, which included consultant obstetricians, non-consultant hospital doctors, and midwives. A second, reminder questionnaire was sent.

### Survey of postnatal women

#### Results

The response rate for the maternal survey was 60.0%. Of 198 completed surveys, 55.1% (*n* = 109) were vaccinated against influenza. Of the 104 women who responded, 7.7% (*n* = 8) were vaccinated during their first trimester, 71.2% (*n* = 74) during their second and 21.2% (*n* = 22) during their third trimester.

Table 1 outlines maternal characteristics and vaccination status. Table 2 presents the results of the stepwise logistic regression analysis. Receiving information was a key determinant of uptake. Associations were also found between vaccination status and socioeconomic status, education level and influenza vaccination in a previous pregnancy.

**Table 1:** Maternal sociodemographics among vaccinated and unvaccinated women (*N* = 198)

	Total responses, n	Vaccinated, *n* (%) (*n* = 109; 55.1%)	Unvaccinated, *n* (%) (*n* = 89; 44.9%)	OR	95% CI
**Mean age at delivery (SD)**	190	34 (4.9)	33.2 (5.8)		
**Age at delivery**	190				
<20 years		1 (1.0)	2 (2.4)	0.56	0.04 to 7.2
20–24 years		6 (5.7)	5 (5.9)	1.3	0.3 to 5.9
25–29 years		9 (8.6)	10 (11.8)	1	–
30–34 years		30 (28.6)	31 (36.5)	1.08	0.38 to 3.0
35–39 years		49 (46.7)	25 (29.4)	2.17	0.78 to 6.0
≥40 years		10 (9.5)	12 (14.1)	0.9	0.27 to 3.17
**Maternal socioeconomic group**	184				
Professional, manager, employer		57 (55.9)	25 (30.5)	1	–
Home duties		8 (7.8)	11 (13.4)	0.32	0.11 to 0.89
Non-manual		28 (27.5)	36 (43.9)	0.34	0.17 to 0.67
Manual		2 (2.0)	3 (3.7)	0.29	0.05 to 1.86
Unemployed		3 (2.9)	5 (6.1)	0.26	0.06 to 1.19
Non-classifiable		4 (3.9)	2 (2.4)	0.87	0.15 to 5.1
**Nationality**	198				
Non-Irish		18 (16.%)	21 (23.6)	1	–
Irish		91 (83.4)	68 (76.4)	1.56	0.77 to 3.15
**Marital status**	198				
Not married		31 (28.4)	34 (38.2)	1	–
Married		78 (71.6)	55 (61.8)	1.55	0.86 to 2.82
**Nulliparous**	198				
No		64 (58.7)	62 (69.7)	1	–
Yes		45 (41.3)	27 (30.3)	1.6	0.89 to 2.92 -
**Obstetric care**	198				
Private or semi-private		65 (59.6)	34 (38.2)	1	–
Public		44(40.4)	55 (61.8)	0.42	0.24 to 0.74
**Smoking status**	197				
Non-smoker		101 (92.7)	73 (83.0)	1	–
Smoker		8 (7.3)	15 (17.0)	0.38	0.15 to 0.94
**Comorbidity^a^**	193				
No		84 (77.1)	72 (85.7)	1	–
Yes		25 (22.9)	12 (14.3)	1.79	0.84 to 3.8
**Level of completed education**	193				
University degree		69 (64.5)	42 (48.8)	1	–
Non-university higher-level course		17 (15.9)	23 (26.7)	0.45	0.22 to 0.94
School completion exam or equivalent		17 (15.9)	14 (16.3)	0.74	0.33 to 1.65
Mid-school exam or equivalent		4 (3.7)	7 (8.1)	0.35	0.09 to 1.26
**Vaccinated in previous pregnancy**	125				
No		23 (35.4)	48 (80.0)	1	–
Yes		42 (64.6)	12 (20.0)	7	3.24 to 16.44
**Received information on vaccine this pregnancy**	194				
No		5 (4.6)	42 (48.8)	1	–
Yes		103 (95.4)	44 (51.2)	19.6	7.29 to 53.0
**Healthcare provider advised vaccination**	194				
No		10 (9.3)	40 (46.0)	1	–
Yes		97 (90.7)	47 (54.0)	8.25	3.8 to 17.93
**Healthcare provider offered vaccination**	193				
No		18 (17.0)	56 (64.4)	1	–
Yes		88 (83.0)	31 (35.6)	8.83	4.5 to 17.23

^a^Comorbidity for which influenza vaccination is indicated, for example chronic heart disease, chronic liver disease, chronic renal failure, chronic respiratory disease, chronic neurological disease, diabetes mellitus, Down syndrome, haemoglobinopathies, morbid obesity, immunosuppression due to to disease or treatment, children aged ≥6 months with any condition that can affect lung function, or on long-term aspirin therapy (because of the risk of Reyes syndrome).^18^

CI = confidence interval. OR = odds ratio.

**Table 2.** Multivariate analysis of vaccination status among postnatal women (*N* = 198)

	Total responses, *n*	aOR^a^	95% CI
**Socioeconomic status**	190		
Professional, manager, or employer		1	–
Not-professional, manager, or employer		0.29	0.01 to 0.89
**Education level**	195		
University		3.69	1.04 to 13.09
Non-university		1	–
**Vaccinated in previous pregnancy**	125		
Yes		5.26	1.69 to 15.62
No		1	–
**Vaccination information received**	194		
Yes		12.8	2.65 to 62.5
No		1	–
**Healthcare worker offered vaccination**	193		
Yes		2.74	0.89 to 8.4
No		1	–

^a^Odds ratios adjusted for socioeconomic status, education level, vaccinated in previous pregnancy, whether vaccination information was received, and whether the healthcare worker offered vaccination.

Both vaccinated and unvaccinated women were asked details of their sources of information on vaccination. Of the 147 responders that received information about vaccination, 87.1% (*n* = 128) were informed by an HCP, 21.1% (*n* = 31) were informed by 'word of mouth', 20.4% (*n* = 30) read literature on the topic, 10.9% (*n* = 16) accessed websites and online resources, and 8.8% (*n* = 13) were informed at antenatal classes.

**Figure 1.**
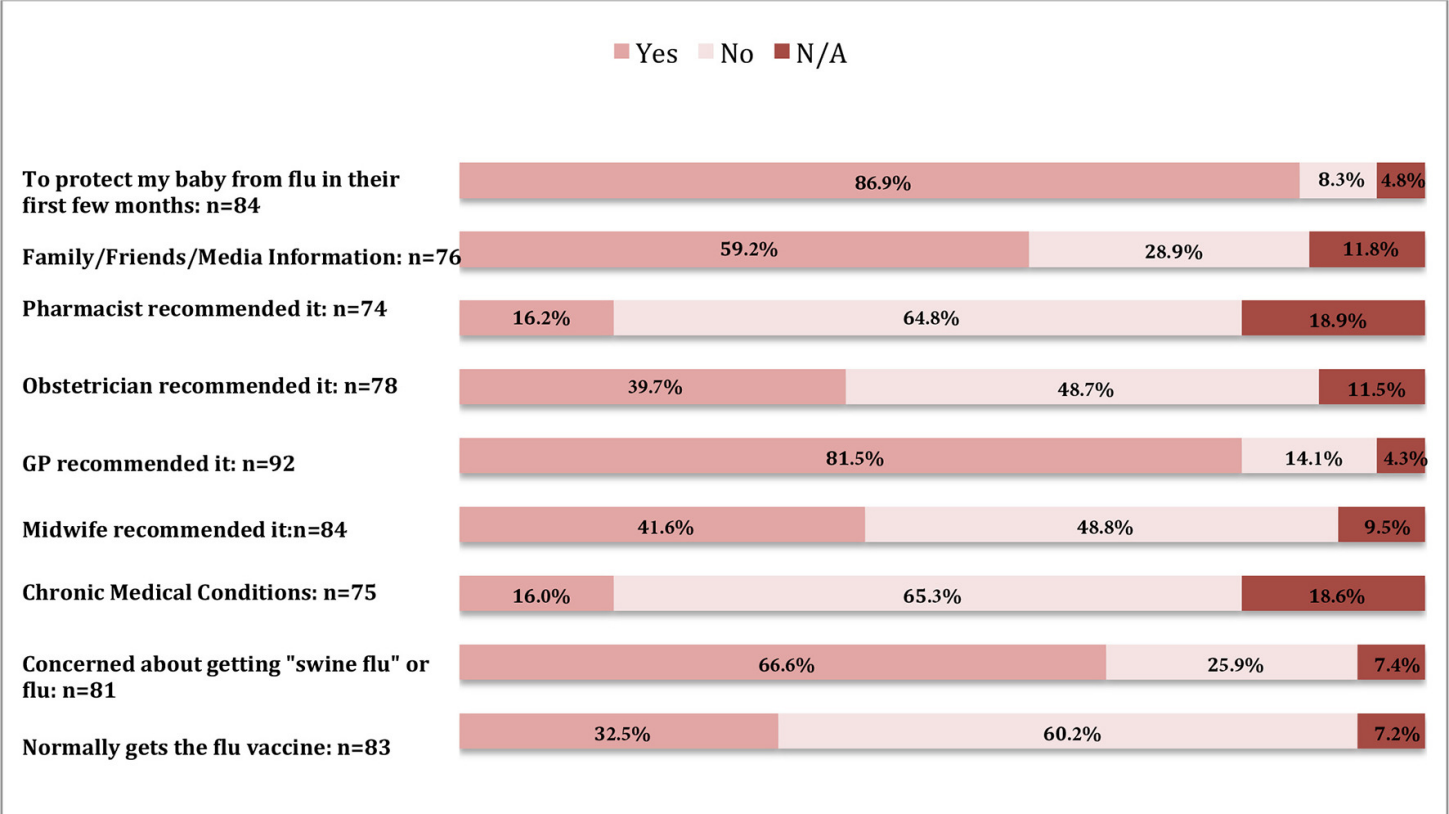
Reasons for receiving the influenza vaccine.

Figure 1 illustrates the reasons vaccinated women cited for getting the flu vaccine. The most common reasons for vaccination were to protect their baby from flu (86.9%; *n* = 73), and the responder's GP recommended it (81.5%; *n* = 75).

**Figure 2.**
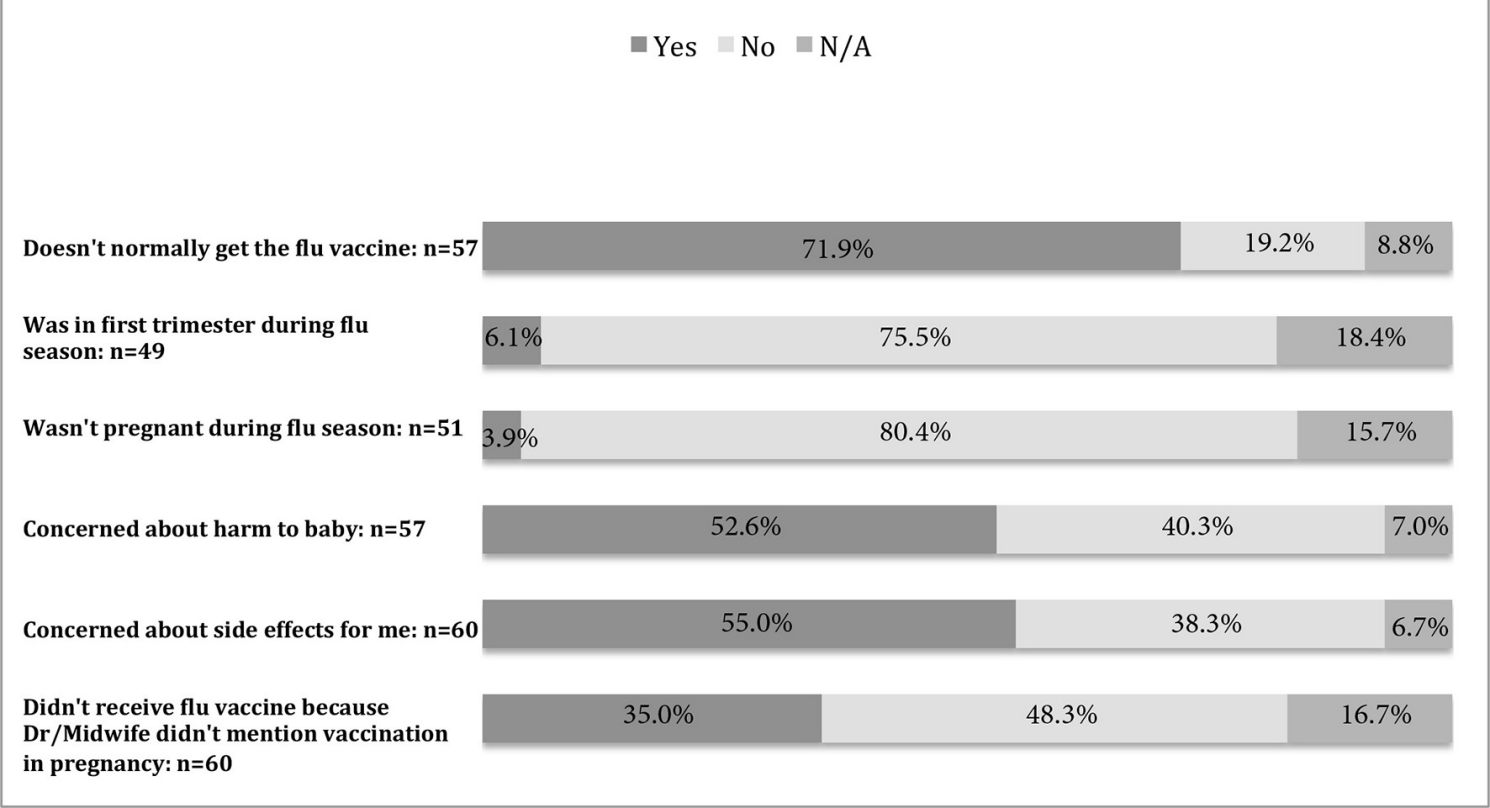
Reasons for not receiving the influenza vaccine

Women were asked their reasons for not receiving the flu vaccine during pregnancy. The most common reason for not being vaccinated, among 71.9% of responders (*n* = 41) was 'Doesn’t normally get the flu vaccine'. Of the unvaccinated responders, 52.6% (*n* = 30) were concerned about harm to baby and 55.0% (*n* = 33) were concerned about harm to themselves, as demonstrated in Figure 2.

With regard to access to information, 84.6% (*n* = 159) of responders agreed that they had sufficient access to information on vaccination in pregnancy. The majority of responders (79%; *n* = 150) agreed that influenza during pregnancy puts women at a greater risk of illness than non-pregnant women.

Of 108 responders that reported receiving the vaccine, 65.7% (*n* = 71) were vaccinated by their GP, 12.0% (*n* = 13) by a pharmacist, 2.7% by a midwife (*n* = 3), and 0.9% (*n* = 1) by a hospital doctor. Those listed as 'other' included those vaccinated by occupational health services (*n* = 11) and nurses in GP surgeries (*n* = 9).

The pertussis vaccine is recommended for administration in each pregnancy to protect pregnant women and their infant, as early as possible between 16–36 weeks’ gestation.^19^ It is administered as part of the Tdap vaccine (tetanus, diphtheria, and acellular pertussis). Responders were asked whether they were vaccinated against pertussis during pregnancy and of 198 responders, 32.3% (*n* = 64) were vaccinated.

### HCP survey

#### Results

##### Knowledge of vaccination

Of 5837 disseminated surveys, 20.2% (*n* = 1180) were completed; 732 GPs, 419 pharmacists, and 29 hospital workers responded to the survey. The majority of responders were female (69.5% ; *n* = 820). The largest group of responders were in the 35–44 year age group (37.6%; *n* = 444), and 44.3% (*n* = 523) of responders were 15 years qualified. Eight hundred and nineteen (69.4%) responders were vaccinated. Eight hundred and four (68.1%) responders correctly answered all questions assessing knowledge of influenza vaccination in pregnancy. A key objective of the HCP survey was to assess knowledge of the risks associated with influenza during pregnancy. One thousand and eleven (86.1%) responders strongly agreed or agreed that influenza increased risk of maternal complications, with a significant difference between HCP groups, with GPs and hospital-based HCPs answering more accurately than pharmacists (*P* = 0.003). Of 1172 responders, 74.8% (*n* = 877) strongly agreed or agreed that influenza was associated with increased risks of fetal complications.

##### Consequences of influenza infection during pregnancy

Fewer responders correctly answered all questions assessing knowledge of maternal and fetal consequences of infection. This ranged from 42.7% (*n* = 177) of responding pharmacists, to 62.1% (*n* = 18) among hospital staff. Significant differences in knowledge of consequences of infection were noted by HCP role (*P* = 0.001) and by HCP vaccination status (*P* = 0.004).

Overall, HCPs were less knowledgeable about the potential consequences of infection on the developing fetus (52.1%; *n* = 611 had correct knowledge) — which include preterm delivery, miscarriage, and fetal death — when compared with their knowledge of maternal consequences, which include increased hospitalisation, increased intensive care unit admission, and maternal death (84.5%; *n* = 995 had correct knowledge).

##### Recommendations for vaccination

Overall knowledge of vaccination recommendations was good, with 97.6% of responders (*n* = 1144) correctly identifying that vaccination was recommended for all pregnant women during the flu season.

##### Responsibility for vaccination

More GPs felt that it was their role to discuss vaccination (92.9%; *n* = 676) than any other HCP, and to offer to vaccinate pregnant women (91.7%; *n* = 666).

##### Safety of vaccination

HCPs were asked whether they felt confident recommending vaccination in pregnancy, as they believed it to be safe in pregnancy; in total, 12.5% (*n* = 146) of HCPs who responded either disagreed (2.1%; *n* = 25), strongly disagreed (1.4%; *n* = 17), or neither agreed nor disagreed (8.9%; *n* = 104) with this statement. Significant differences were noted by years qualified, with those >15 years qualified reporting less confidence in vaccine safety. HCPs who were unvaccinated themselves were more likely to disagree with the statement on vaccine safety during pregnancy.

##### Factors encouraging vaccination

HCPs were asked their opinion on factors that may encourage vaccination. Recommendations from the patient’s obstetrician scored highest overall, at 89.0% (*n* = 1050).

##### HCP vaccination

At the time of completing the questionnaire, 69.6% (*n* = 819) of HCPs had received the influenza vaccination. Those <5 years post-qualification showed the lowest rates of influenza vaccination uptake.

##### Vaccination recommendation

Regarding HCPs recommendations of the influenza vaccine to pregnant women, 73.2% (*n* = 532) of responding GPs, 72.4% (*n* = 21) of hospital based HCPs, and 40.1% (*n* = 158) of responding pharmacists report that they recommend influenza vaccination all of the time.

##### Access to vaccination

Of all three HCP groups, GPs were the most popular choice to give the vaccine at 59.0% (*n* = 696). Furthermore, 61.6% of pharmacists (*n* = 258) and 58.6% (*n* = 17) of hospital-based HCPs took the view that the vaccine should be given in any location.

##### Pertussis vaccination

The majority of responding HCPs (75.6%; *n* = 885) correctly answered that pertussis vaccination was recommended during pregnancy. Of 1160 responders, only 32.4% (*n* = 376) strongly agreed and 29.7% (*n* = 344) agreed with the statement that they felt confident recommending pertussis vaccination, as they believed it to be safe during pregnancy.

## Discussion

### Summary

The study found that 55.1% of women had received the influenza vaccine during their pregnancy. Just 32.3% of women were vaccinated against pertussis during pregnancy.

This study has identified that women with higher socioeconomic status, those who attained a university degree, and who attended as a private or semi-private patient, were more likely to be vaccinated against influenza during pregnancy. A key determinant of vaccination in pregnancy was receiving information from a health professional.

Most HCPs that responded were aware that influenza vaccination should be offered to all pregnant women, and could be given in any trimester.

Perceived responsibility for vaccination was weighted towards GPs. Those HCPs who, themselves, were vaccinated were more likely to assume responsibility for discussing vaccination.

### Strengths and limitations

This study was strengthened by the diversity of patients included in the study. The Rotunda Hospital cares for public, semi-private, and private patients. The setting of this maternity hospital in Dublin’s city centre means that its patients have diverse sociodemographic backgrounds.

Non-response bias may have led to an overestimation of vaccine uptake. Convenience sampling may also have introduced bias, with more semi-private and private patients participating.

The women’s partners' vaccination status was not routinely recorded in the survey, which may have added further understanding to the factors that influence a decision to be vaccinated.

To facilitate an optimal response, the HCP questionnaire took just 3 minutes to complete. This limited the depth and breadth of questions on the survey. Not all HCPs who responded may be directly involved in the care of pregnant women.

### Comparison with existing literature

Maternal sociodemographic characteristics may influence the vaccination status of pregnant women. Maternal characteristics of unvaccinated women were consistent with the findings of similar national and international studies.^7–8,16,20^

Those that had good knowledge surrounding vaccination during pregnancy were more likely to be vaccinated. Other studies have reported immunisation rates to be lower for women who did not access medical or official information sources.^21^

These findings indicate that a high percentage of women are concerned about the safety profile of the influenza vaccine during pregnancy. Other international studies have reported the safety concerns of women regarding the influenza vaccine during pregnancy and breastfeeding.^22^ Both Irish and international guidelines recommend the flu vaccine for all women that are or will be pregnant during flu season.^4,23–24^

The recommendation from an HCP to receive the flu vaccine during pregnancy is a key determinant of vaccine uptake. Previous international studies reported similar findings.^9,10,12^ Advising and offering to vaccinate women has been associated with a higher uptake of vaccination during pregnancy.^25^

This study demonstrated good knowledge among HCPs of the possible risks that influenza presents to the mother. Knowledge of potential consequences on the developing fetus among HCPs was poor. Limited knowledge and poor perception of the risks of influenza in pregnancy among HCPs may act as a potential barrier to vaccine uptake.^26–27^

Similar to the findings of a UK study, GPs were significantly more likely to assume responsibility for vaccination than other HCP groups.^28^ HCPs that lack confidence in vaccine safety are more likely to allow the decision to be directed by the women herself.^27^ HCPs are more likely to recommend vaccination to pregnant women if they themselves had been vaccinated.^13^

### Implications for research and practice

Uptake of vaccination during pregnancy is sub-optimal and may relate to pregnant women’s concerns relating to the safety of vaccines during pregnancy. These concerns should be addressed in a systematic way to improve vaccination uptake.

Elements of this study may be repeated as future audits, to assess temporal uptake trends of vaccination, as the results are reproducible and in line with the findings of similar studies.

HCP education and support may be required to address knowledge gaps relating to vaccination during pregnancy. The sociodemographic determinants of a woman’s vaccination status should be considered in targeting future health promotion campaigns. All health professionals have a role in providing information on the benefits and safety of vaccination in pregnancy. Vaccines should be available in a range of locations.
